# Multi-Omics Sequencing Dissects the Atlas of Seminal Plasma Exosomes from Semen Containing Low or High Rates of Sperm with Cytoplasmic Droplets

**DOI:** 10.3390/ijms26031096

**Published:** 2025-01-27

**Authors:** Zilu Zhang, Xiaoxian Xu, Fumei Chen, Qingyou Liu, Zhili Li, Xibang Zheng, Yunxiang Zhao

**Affiliations:** 1 College of Animal Science & Technology, Guangxi University, Nanning 530004, China; ziluzhang2022@163.com (Z.Z.); 2118301037@st.gxu.edu.cn (X.X.); fumeichen@gxu.edu.cn (F.C.); 2 College of Animal Science and Technology, Foshan University, Foshan 528231, China; qyliu-gene@fosu.edu.cn (Q.L.); lizhili@fosu.edu.cn (Z.L.)

**Keywords:** cytoplasmic droplets, exosomes, proteomics, transcriptomics

## Abstract

Sperm cytoplasmic droplets (CDs) are remnants of cytoplasm that can cause a number of problems if it not shed from the sperm after ejaculation. Exosomes can rapidly bind to sperm, but it is not clear whether exosomes can affect the migration and shedding of CDs. We first extracted and characterized seminal plasma exosomes from boar semen containing sperm with low or high rates of CDs. Then, the transcriptomic and proteomic detection of these exosomes were performed to analyze the differences between the two groups of seminal plasma exosomes. The results revealed that 486 differentially expressed genes (DEGs), 40 differentially expressed proteins (DEPs), and 503 differentially expressed lncRNAs (DElncRNAs) were identified between the low CD rate group and high CD rate group. Integrative multi-omics analysis showed that exosome components may affect migration and shedding of cytoplasmic droplets by influencing cytoskeletal regulation and insulin signaling, including regulation of the actin cytoskeleton, ECM–receptor interaction, axon guidance, insulin secretion, and the insulin signaling pathway. Overall, our study systematically revealed the DEGs, DEPs, and DElncRNAs in seminal plasma exosomes between low CD rate semen and high CD rate semen, which will help broaden our understanding of the complex molecular mechanisms involved in the shedding of CDs.

## 1. Introduction

Cytoplasmic droplets (CDs) are formed in the testicular spermatogenic epithelium. CDs are the cytoplasmic residue of round spermatid cytoplasm that has been phagocytosed by Sertoli cells [1]. The cytoplasmic droplet is a marker of normal sperm morphology and plays an important role in the maturation of epididymal sperm [2].

One of the earlier findings of CDs was that CDs move in a peristaltic-like manner along the midpiece of the flagellum from the neck to the annulus as spermatozoa pass through the epididymal duct [3]. Current research suggests that CDs are a temporary source of energy for sperm as they mature in the epididymis, and they can exchange small RNAs (mainly tsRNA and rsRNA) and proteins with the sperm [4,5,6,7]. This is bound to have a direct effect on the sperm. However, if CDs are not shed during ejaculation, they can interfere with the morphology and function of sperm. CDs remaining on the sperm will increase the osmotic pressure inside the sperm, causing it to absorb water, swell, and rupture or form a corner; it also disrupts sperm motility [1]. In severe cases, it can lead to male sterility [1].

Many surveys show that CD retention is considered to be the most common abnormal sperm morphology in boar semen and the main reason for reducing the utilization rate of boar semen [8,9,10,11]. This is because the retention of CDs on ejaculated spermatozoa reduces the female’s pregnancy rate, delivery rate, and litter size [12,13,14]. Environmental conditions, nutritional conditions, and age can affect CDs [15,16]. Boars living in environments with high temperature and humidity have higher residual rates of CDs [15]. It has also been shown that the deletion of *sperm1*- and *15-lipoxygenase* genes causes the CDs to fail to drop off the sperm in mice [17,18]. New research has revealed that the *SYPL1* gene is enriched in the cytoplasmic droplets of sperm, and its absence results in the failure to produce sperm protoplasmic droplets, leading to a significant reduction in fertility in mice [19].

Semen plasma is the liquid part of semen excluding cells, which constitutes the survival and maturation environment of sperm and regulates the movement and morphology of sperm [20]. The seminal plasma contains a large number of lipid particles. Among them, the major components of seminal exosomes are prostasomes and epididymosomes that are secreted by the prostate and epididymis, respectively [21]. The exosomes in the seminal plasma are very important for sperm motility, morphology, acrosome response, capacitation, and fertilization [22,23,24]. They could alter the lipid composition of the sperm membrane and assist in the production of future sperm motility and the ability to penetrate the zona pellucida [25]. Meanwhile, ATP generated in seminal plasma exosomes can finely modulate mitochondrial metabolism to regulate sperm motility [26]. We previously analyzed boar seminal plasma exosomes from semen containing spermatozoa with or without CDs and identified 16 significantly different miRNAs [27]. This suggests that exosomes in seminal plasma may have an effect on sperm CDs. At present, no other relevant reports have been reported, and it not clear how the exosomes in the seminal plasma can affect the shedding of sperm CDs.

Profiling exosomal proteins, mRNAs, and lncRNAs can be helpful for the identification of molecular markers for diagnosis and prognosis and for closure of knowledge gaps regarding the shedding of CDs. In this study, we performed a multi-omics analysis on the cargos in exosomes from semen containing sperm with high or low rates of CDs to systematically elucidate the biological processes related to sperm CDs. The results of this study may help to generate new perspectives on the shedding of sperm CDs and ultimately provide a new scheme for improving the quality and utilization rate of boar semen.

## 2. Results

### 2.1. Characterization of Exosomes Derived from Seminal Plasma

**Figure 1:**
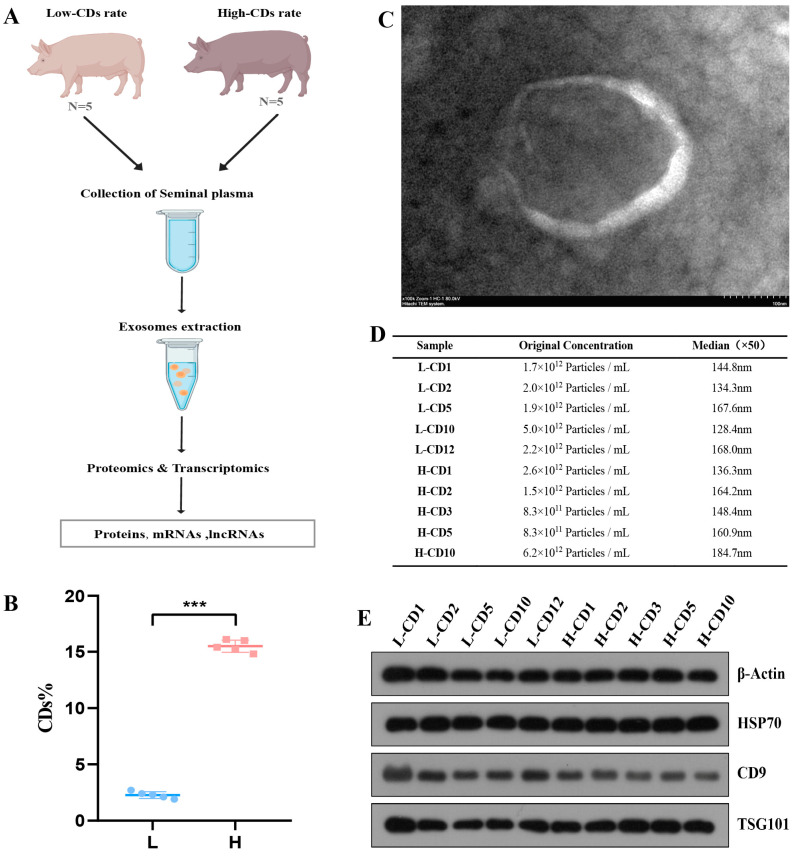
**Characterization of exosomes.** (**A**) Overview of the experimental design for multi-omics analysis. (**B**) Statistical analysis of CD rate for the samples. *** *p* < 0.001. (**C**) The typical morphological characteristics of isolated exosomes were detected using TEM. (**D**) The size distribution of serum exosomes was determined using NTA. (**E**) Membrane proteins of exosome were detected using Western blot.

A schematic procedure for the study is shown in Figure 1A. Boar seminal plasma was obtained from semen containing sperm with low or high rates of cytoplasmic droplets, and exosomes were isolated from the seminal plasma. The CD rates were all less than 3% in the low group and more than 14% in the high group (Figure 1B). A DIA-based proteomics strategy and high-throughput sequencing approach were used to quantitate exosome cargos, including proteins, mRNAs, and lncRNAs.

To characterize the exosomes, we performed TEM, NTA, and immunoblotting. The TEM findings revealed that exosomes had the usual cup shape (Figure 1C). The NTA findings revealed that the concentration of isolated exosomes was 1.9 × 10^12^ particles/mL and that exosomes were between 50 and 150 nm in diameter, which is compatible with the reported exosome size (Figure 1D). In addition, the immunoblotting results demonstrated that the vesicles were positive for markers of exosomes, including HSP70, TSG101, and CD63 proteins (Figure 1E).

### 2.2. Transcriptome Profile of Seminal Plasma Exosomes from Semen Containing Low or High Rates of Sperm with CDs

**Figure 2:**
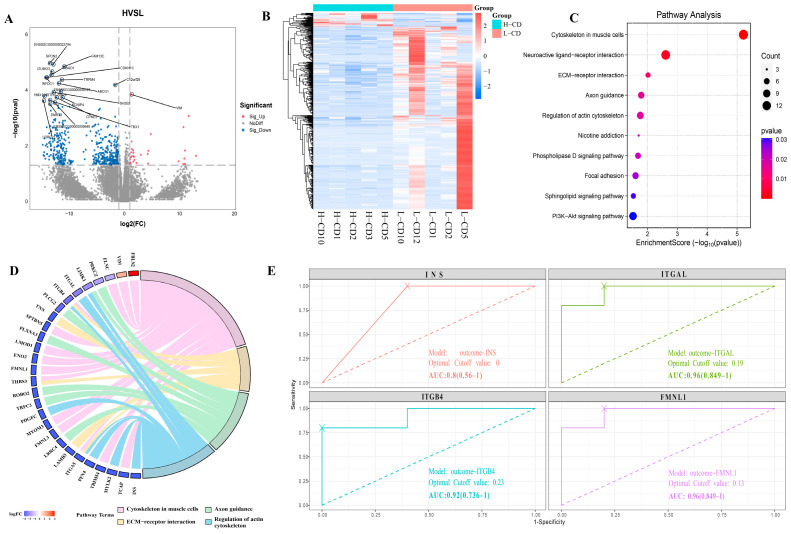
**Seminal plasma exosome DEGs in semen containing sperm with low or high rates of CDs.** (**A**) Volcano map of 486 DEGs between the high CD rate group and low CD rate group. (**B**) Heatmap of DEGs. (**C**) KEGG pathway enrichment analysis of DEGs between the high CD rate group and low CD rate group. The top 10 significant pathways are displayed. (**D**) DEGs enriched in the ECM and cytoskeletal pathways, and trends in the expression of these genes. (**E**) ROC analyses of verified 4 exosomal mRNAs. ROC, receiver operator characteristic; AUC, area under the ROC curve.

The differentially expressed genes (DEGs) were identified between two groups according to the cutoff threshold of |log2FC| ≥ 1 and *p* < 0.05. Compared to the low rate of CD group, the seminal plasma exosomes of the high group contained 486 DEGs, of which 33 were up-regulated, and 453 were down-regulated (Figure 2A). A heatmap shows significantly different transcriptomic patterns of DEGs between the two groups (Figure 2B). These DEGs were mainly enriched in multiple pathways of interest, including cytoskeleton in muscle cells, regulation of actin cytoskeleton, ECM–receptor interaction, and axon guidance pathways (Figure 2C,D). Moreover, a small number of DEGs were enriched in the phospholipase D signaling pathway and PI3K–Akt signaling pathway (Figure 2C). It indicated that all of these differentially enriched pathways may be involved in the progression of CD shedding, but the regulation of cytoskeleton may play a crucial role. Interestingly, the down-regulation of the insulin gene *INS*, which was involved in the above functions, may directly affect insulin signaling (Figure 2D). Consequently, we performed ROC analyses and calculated area under the curve (AUC) of related DEGs to verify their potential. The AUCs for *ITGAL*, *ITGB4*, *FMNL1*, and *INS* were 0.96, 0.92, 0.96, and 0.8, respectively, indicating that these cytoskeleton-related DEGs can be used as markers to determine whether boar spermatozoa have high or low rates of residual cytoplasmic droplets (Figure 2E).

### 2.3. lncRNAs Profile of Seminal Plasma Exosomes from Semen Containing Low or High Rates of CDs

**Figure 3:**
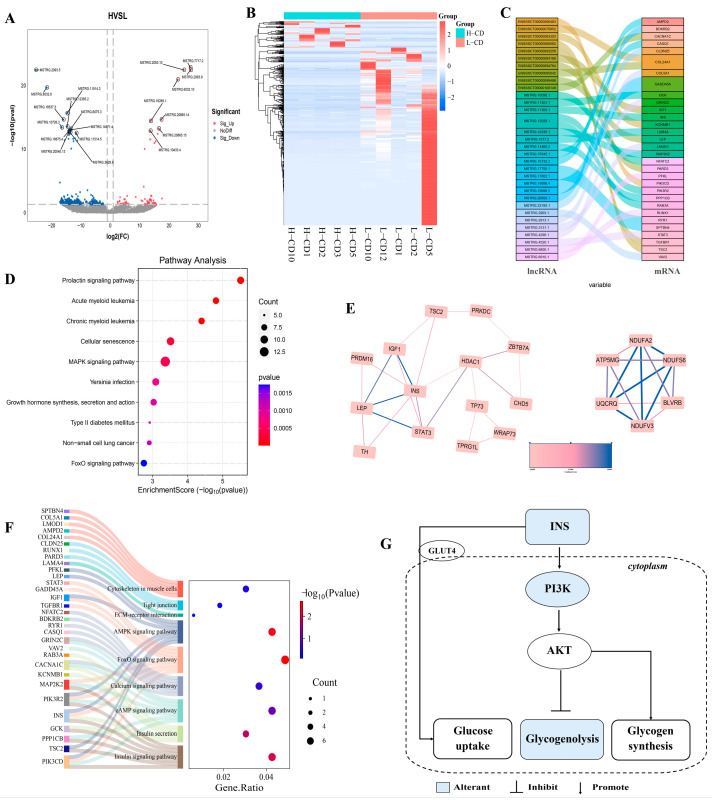
**lncRNAs profile of seminal plasma exosomes in semen containing low or high rates of sperm with CDs.** (**A**) Volcano map of 503 DElncRNAs between the high CD rate group and low CD rate group. (**B**) Heatmap of DElncRNAs. (**C**) Prediction of target genes for DElncRNAs. (**D**) KEGG pathway enrichment analysis of DElncRNAs target genes. The top 10 significant pathways are displayed. (**E**) Core interaction network modules for the target genes of DElncRNAs. (**F**) Demonstration of target genes enriched in key pathways. (**G**) Schematic diagram of the insulin signaling pathway. In the figure, the relevant genes in the pathway represented in blue are the target genes of the DElncRNAs.

After assembling the reads using transcript assembly software, known mRNAs and transcripts smaller than 200 bp were removed. Then, the remaining new transcripts were subjected to coding ability prediction using the prediction software CPC2 (Coding Potential Calculator) (http://cpc2.cbi.pku.edu.cn, 4 June 2024, v2.0) and CNCI (Coding-Non-Coding Index) (https://github.com/www-bioinfo-org/CNCI#install-cnci, 4 June 2024, v1.0). CPC and the CNCI predicted the intersection of transcripts with no coding potential as the final new predicted lncRNAs (Figure S1A). Next, according to the cutoff threshold of |log2FC| ≥ 1 and *p* < 0.05, we identified 503 lncRNAs that were dysregulated in exosomes (Figure 3A,B). To elucidate the potential functions of these differentially expressed lncRNAs (DElncRNAs), we performed KEGG pathway analysis of their target genes, and the top 10 significant pathways are displayed (Figure 3C,D). As shown in Figure 3D, the prolactin signaling pathway, cellular senescence, MAPK signaling pathway, and FoxO signaling pathway were significantly enriched.

To further elucidate the potential relationships among the target genes, we constructed a molecular interaction network and identified the core modules of this network using the MCODE plugin in Cytoscape (https://cytoscape.org, 1 September 2023, v3.10.1). As shown in Figure 3E, we identified two hub clusters. Noteworthy, one of them is the regulatory network of the insulin gene *INS*. We further found that many target genes were involved in insulin-related pathways, such as the insulin signaling pathway, insulin secretion, and the FoxO signaling pathway, which indicates that insulin signaling plays a role in the regulation of CD shedding (Figure 3F,G).

### 2.4. Proteins Profile of Seminal Plasma Exosomes from Semen Containing Low or High Rates of Sperm with CDs

**Figure 4:**
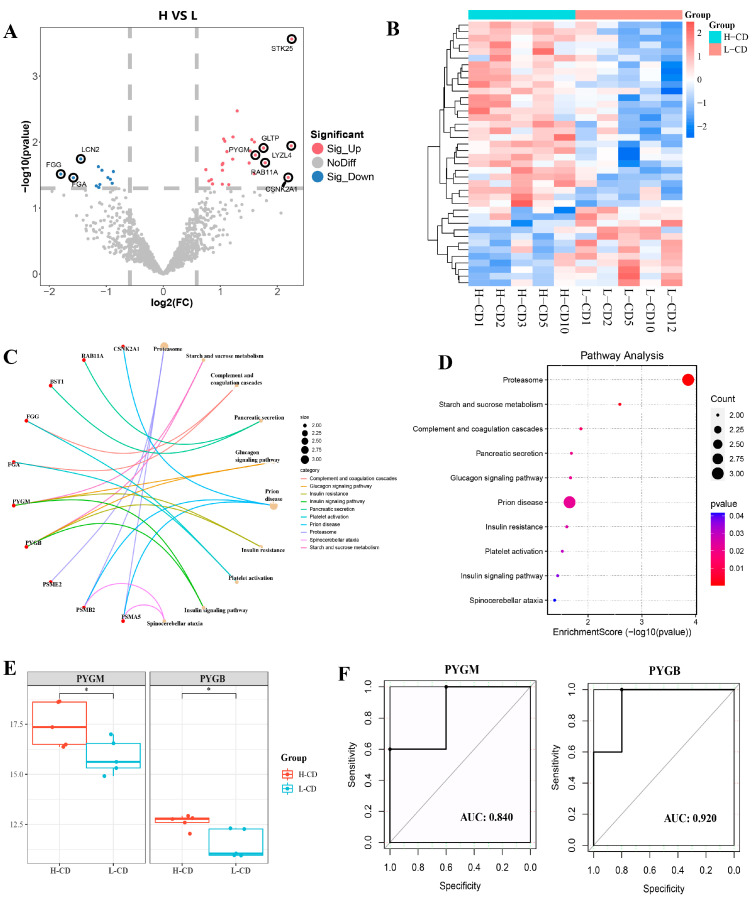
**Seminal plasma exosome DEPs in semen containing low or high rates of sperm with CDs.** (**A**) Volcano map of 40 DEPs between the high CD rate group and low CD rate group. (**B**) Heatmap of DEPs. (**C**) DEPs enriched in the pathways. (**D**) KEGG pathway enrichment analysis of DEPs between the high CD rate group and low CD rate group. The top 10 significant pathways are displayed. (**E**) The expression levels of PYGM and PYGB. * *p* < 0.05 (**F**) ROC analyses of verified PYGM and PYGB proteins.

The differentially expressed proteins (DEPs) were identified between two groups according to the cutoff threshold of |log2FC| ≥ 0.58 and *p* < 0.05. Compared to the group with a low rate of CDs, the seminal plasma exosomes of the group with a high rate contained 40 DEPs, of which 28 proteins were up-regulated, and 12 proteins were down-regulated (Figure 4A, Table S2). Further hierarchical heatmap showed the relative expression characteristics of different proteins between the two groups (Figure 4B). These DEPs were mainly enriched in proteasome, starch and sucrose metabolism, insulin resistance, and the insulin signaling pathway (Figure 4C,D). Among them, glycogen phosphorylases PYGM and PYGB, which are essential enzymes for glycogen degradation, were significantly upregulated in the groups with a high rate of CDs (Figure 4E). It is well known that when insulin decreases, the breakdown of glycogen increases. The RNA of the insulin gene *INS* was significantly down-regulated in the high group, which inhibited insulin signaling. This may also be the reason for the up-regulation of PYGM and PYGB. The AUC values of PYGM and PYGB were 0.84 and 0.92, respectively, indicating that these proteins may be potential diagnostic markers for CDs residues (Figure 4F).

### 2.5. Integrative Analysis of Proteomics and Transcriptomics Datasets Derived from the Seminal Plasma Exosomes

**Figure 5:**
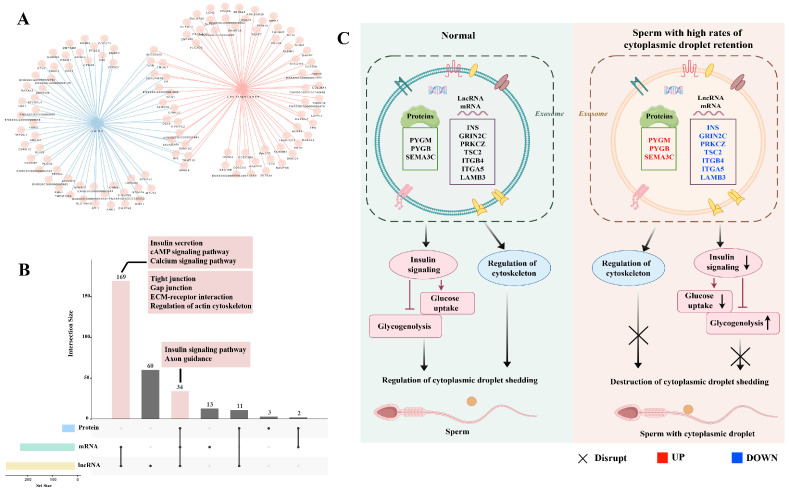
**Integrative multi-omics data analysis, including DEGs, DEPs, and DElncRNAs.** (**A**) Venn analysis of DEGs, DEPs, and DElncRNA. (**B**) Venn analysis of KEGG pathways from DEGs, DEPs, and DElncRNA. (**C**) We hypothesize that exosomes are involved in the migration and shedding of sperm cytoplasmic droplets by acting on cytoskeleton and insulin signaling. Blue color represents down-regulation of expression in the high residue group, red color represents up-regulation of expression in the high residue group (by Figdraw).

The multi-omics analysis results revealed no common genes among the DEGs, DEPs, and DElncRNA target genes (Figure S1B). There are 17 common genes between DEGs and DElncRNA target genes, including the insulin gene *INS* (Figure 5A). We further analyzed the functional changes and found that the KEGG function of DEPs could be almost completely included by the DEGs and DElncRNA target genes, and insulin signaling pathway and axon guidance were the common functions of the three (Figure 5B). At the same time, we also found other insulin-related functions in DEGs and DElncRNA target genes, such as insulin secretion. This also indicates that insulin signal transduction plays an important role in the process of protoplasmic droplet shedding, and its mechanism needs to be further studied.

We also found other common functions in DEGs and DElncRNA target genes, such as cytoskeleton and ECM–receptor interaction (Figure 5B). These functions and axon guidance may directly affect the binding of cytoplasmic droplets and sperm, which needs to be further explored. Based on the above results, we hypothesize that exosomes from seminal plasma may affect cytoplasmic droplet shedding by acting on the insulin signaling pathway and cytoskeletal regulation (Figure 5C).

## 3. Discussion

The molecular mechanism of protoplasmic droplet shedding is still unknown. A number of studies in animals and humans have been implemented to determine the compositions and roles of semen extracellular vesicles [28]. Recent studies have indicated that exosomes, as semen-derived extracellular vesicles, are rapidly absorbed by the sperm plasma membrane and play a crucial role in sperm structure and function [29,30,31,32]. Systematic studies on the components in seminal plasma exosomes from semen containing sperm with high rates of CDs will be helpful for elucidating the functions and related regulatory mechanisms of exosomes. In the present study, we performed integrative proteomics and transcriptomics analyses to assess the landscape and the molecular signatures of exosomes from semen containing sperm with high rates of CDs and to promote a new understanding of CD shedding. Due to the source of available samples, only *Duroc* boars were selected as the research target in this study.

Using multi-omics analysis of the components, we found that many DEG and LNC target genes are enriched in pathways such as ECM–receptor interaction and cytoskeletal regulation, with genes such as *VIM* and integrins *ITGA5* and *ITGB4* significantly down-regulated in the high residue group. The extracellular matrix (ECM) is part of the cytoskeleton. In addition to maintaining cellular morphology, the ECM interacts with cells to regulate a variety of functions, including cell junctions, adhesion, migration, and differentiation [33,34]. Integrins are the main adhesion receptors to ligands of the ECM, linking the actin cytoskeleton to the ECM and enabling cells to sense matrix rigidity and mount a directional cell migration response to stiffness gradients [35,36]. Along with changes in integrins, the actin cytoskeleton and cytoskeleton must also be altered, and we found the expression of cytoskeleton-related genes, such as *MYLK2*, *FLNC*, and *TCAP*, were also significantly reduced in the high residue group. Although the role of the ECM and cytoskeletal recombination in spermatogenesis [37], capacitation and acrosomal reactions have been established [38,39]. However, its role in the migration of sperm cytoplasmic droplets is not clear yet. In addition, the internal organellar membranes rotate in a vortex-like manner around the axoneme and mitochondrial sheath as the CD slides along the flagellum. In this process, they appear to alter the plasma membrane of the spermatozoa [1]. This raises the intriguing possibility that exosomes in seminal plasma may influence the migration and shedding of sperm CDs by regulating ECM and cytoskeletal recombination. As far as we know, this is the first time this idea has been put forward, but the specific processes and modes of regulation involved in these functions need to be further explored.

In addition to these results we found that the insulin signaling pathway was significantly enriched in exosomal differential proteins, mRNA, and lncRNA. Importantly, the expression of the insulin gene *INS* was significantly down-regulated in the high residue group, and this may lead to attenuated insulin signaling. Activation of the insulin receptor corresponds to two crucial metabolic functions, i.e., uptake of glucose and storage of glycogen [40,41]. Insulin receptors are involved in the recruitment of phosphatidylinositol-3-kinase (PI3K) in the insulin signaling pathway, which in turn leads to phosphorylation and activation of the serine/threonine kinase Akt (protein kinase B) [42,43,44]. Upon activation of Akt, intracellular vesicles containing glucose transporter protein 4 (GLUT4) are transported to the plasma membrane, thereby allowing the cell to take up glucose [40]. Once glucose is transported into the cells, it gets phosphorylated to form G6P with the help of glucokinase (GCK) [45,46]. We identified *GCK*, *PIK3R2*, and *PIK3CD* as target genes for differential lncRNA in exosomes. At the same time, activation of Akt by insulin causes the phosphorylation and subsequent inhibition of GSK3β. Inactivation of GSK3β leads to the dephosphorylation of glycogen synthase (GS) and increased glycogen synthesis [47]. On the contrary, when insulin levels are low, gluconeogenesis and glycogenolysis are stimulated to maintain the glucose levels [47]. This may also explain the up-regulation of PYGM and PYGB expression in the high residue group. Glucose metabolism plays an important role in spermatogenesis [48]. Glucose metabolism is necessary for the maintenance of basic cell activities and their specific characteristics, such as motility and the activity of a mature sperm that leads to fertilization [49]. Here, we propose a new hypothesis that an imbalance in glucose metabolism caused by reduced insulin levels may affect the migration and shedding of sperm cytoplasmic droplets.

Our study showed that the seminal plasma exosomes may be involved in the migration and shedding of sperm cytoplasmic droplets by acting on cytoskeleton and insulin signaling. However, these conclusions currently lack further functional validation. Further work should validate this with more detailed experiments, determine the role of exosomes on the migration and shedding of cytoplasmic droplets, and clarify the specific molecular mechanism.

## 4. Materials and Methods

### 4.1. Sample Collection

Duroc boars aged 15–28 months were selected for this study. All boars came from the same farm (Guigang City, Guangxi Province, China) and received the same nutrition under the same feeding and management conditions. Boars are kept in controlled environmental conditions with a temperature of 20 °C to 24 °C and a relative humidity of 60%. Semen was collected by personnel, and the frequency of ejaculates was 5–7 days. All the boars on the farm were assessed for semen quality for 1 months. Boars with a CDs residue rate of less than 3% throughout the observation period and at the time of sample collection were included as the low residue group (*n* = 5). Boars with a CDs residue rate of more than 14% throughout the observation period and at the time of sample collection were included as the high residue group (*n* = 5). Semen samples were assessed using the CASA system (IMV Technologies, L’Aigle, France). The cytoplasmic droplet rate in this paper is the sum of the proximal and distal cytoplasmic droplet rates. The test data of all samples are shown in Supplementary Table S1.

After the semen sample was fully liquefied, it was centrifuged at 1500× *g* for 20 min at room temperature to separate the seminal plasma. The seminal plasma was frozen at −80 °C.

### 4.2. Isolation of Exosomes

In our study, ultracentrifugation was used to isolate serum exosomes following the protocol described previously [27]. The seminal plasma was centrifuged at 3000× *g* for 30 min at 4 °C to remove large cell fragments or debris. The supernatant was filtered through a 0.22-μm membrane filter, after which the filtrate was centrifuged at 100,000× *g* for 80 min at 4 °C. The resulting pellet was resuspended in phosphate buffer saline (PBS) on a sucrose cushion and centrifuged at 100,000× *g* for 2 h to pellet the exosomes.

### 4.3. Characterization of Exosomes

The morphology of the isolated exosomes was observed using transmission electron microscopy (TEM) (Hitachi, Tokyo, Japan); the number and size of the exosomes were measured with nanoparticle tracking analysis (NTA) using a ZetaView_Particle Metrix (Particle Metrix, Inning am Ammersee, Germany). Meanwhile, specific markers for exosomes, including HSP70 (Abcam, CBD, Cambridge, UK), CD9 (Abcam, CBD, UK), and TSG101 (Beyotime, Shanghai, China), were detected by Western blot analysis.

### 4.4. RNA Sequencing

Total RNA was extracted using Trizol reagent (Thermo Fisher Scientific, Waltham, MA, USA) following the manufacturer’s procedure. The total RNA quantity and purity were analyzed using a Bioanalyzer 2100 and RNA 6000 Nano LabChip Kit (Agilent, Santa Clara, CA, USA) with RIN number > 7.0. Then, ribosomal RNA (rRNA) was depleted from total RNA with a Ribo-Zero Gold rRNA Removal Kit (Illumina, cat. MRZG12324, San Diego, CA, USA). For preparation of RNA libraries, an NEBNext^®^ UltraTM RNA Library Prep Kit (NEB Cat#E7530L, Ipswich, MA, USA) for lncRNAs was used according to the manufacturer’s instructions. Paired-end sequencing (PE150) was performed for mRNAs and lncRNAs on an Illumina NovaseqTM 6000 sequence platform. Reads obtained from the sequencing machines were further filtered using Cutadapt (https://cutadapt.readthedocs.io/en/stable/, 19 June 2024, v4.9). The raw sequence data were submitted to the NCBI Short Read Archive (SRA) with accession number PRJNA1164465.

The software DESeq2 (https://github.com/thelovelab/DESeq2, 4 June 2024, v1.42.0) were used for differential expression analyses of the RNA-seq raw counts. The genes, mRNAs, and lncRNAs with a parameter of *p* < 0.05 and absolute |log2FC| ≥ 1 were considered differentially expressed mRNAs, and lncRNAs. Differentially expressed coding RNAs were then subjected to enrichment analysis of GO functions and KEGG pathways. To further analyze the key or hub modules, we used the Molecular Complex Detection (MCODE) plugin (v1.5.1) in Cytoscape.

### 4.5. Bioinformatic Analysis of the RNA-Seq Data

Transcripts that overlapped with known mRNAs and lncRNAs and transcripts shorter than 200 bp were filtered. Then, we utilized CPC2 (http://cpc2.cbi.pku.edu.cn, 4 June 2024, v2.0) and CNCI (https://github.com/www-bioinfo-org/CNCI#install-cnci, 4 June 2024, v1.0) with default parameters to predict novel transcripts with coding potential. All transcripts with a CPC score < 0.5 and CNCI score < 0 were retained and considered as novel lncRNAs. The remaining transcripts with class codes (I, j, o, u, x) were considered as novel lncRNAs. The potential target genes affected by cis-regulation were obtained by integrating the data on the differentially expressed lncRNAs and their adjacent (within 100,000 bp) mRNAs.

### 4.6. Proteomics Analysis

A 100 µg aliquot of extracted proteins from each sample was then subjected to reduction. Then, trypsin (trypsin:protein = 1:50) was added, and the sample was incubated at 37 °C overnight. It was then desalted and lyophilized for mass spectrometry analysis. The samples were fractionated using a high pH reverse-phase fractionator and measured in DIA mode. The mass spectrometer was operated on a quadrupole Orbitrap mass spectrometer (Q Exactive HF-X, Thermo Fisher Scientific, Bremen, Germany) coupled to an EASY nLC 1200 ultra-high pressure system (Thermo Fisher Scientific) via a nano-electrospray ion source. For DIA, the acquisition method consisted of one MS1 scan (350 to 1500 *m*/*z*, resolution 60,000, maximum injection time 50 ms, and AGC target 3 × 10^6^) and 42 segments at varying isolation windows from 14 *m*/*z* to 312 *m*/*z* (resolution 30,000, maximum injection time 54 ms, and AGC target 1 × 10^6^). Stepped normalized collision energy was 25, 27.5, and 30. The default charge state for MS2 was set to 3.

A DIA library was used to search the MS data of the single-shot samples in the Spectronaut16 (Biognosys, Zürich, Switzerland) software for final protein identification and quantitation. All searches were performed against the uniprot Sus scrofa SP proteome database (20,627 target sequences downloaded on 19 December 2023).

### 4.7. Statistical Analysis

GraphPad Prism 8.0 was used for statistical analysis. All results are presented as the mean ± standard error of the mean (SEM). Student’s *t*-test was performed for comparison between two groups. Differences were considered statistically significant when the *p* value was <0.05.

## 5. Conclusions

Our study showed that boar sperm with different levels of residual cytoplasmic droplets had different fractions of mRNA, lncRNA, and proteins in their seminal plasma exosomes. We first hypothesize that exosomes are involved in the migration and shedding of sperm cytoplasmic droplets by acting on cytoskeleton and insulin signaling. We also preliminarily screened and identified marker genes and proteins used to distinguish between high and low residual rates of cytoplasmic droplet retention. This study provides a new way to elucidate the molecular mechanism of boar sperm cytoplasmic droplet shedding and reveals a possible new role of seminal plasma exosomes in sperm. It also provides a possible new scheme for reducing the residue of sperm cytoplasmic droplets and improving the utilization rate of boar semen.

## Data Availability

The datasets supporting the conclusions of this article are included within the article and its supplementary file. The RNA-seq data of exosomes have been deposited in the NCBI Sequence Read Archive (https://www.ncbi.nlm.nih.gov/sra/; accession number: PRJNA1164465).

## Supplementary Materials

The following supporting information can be downloaded at: https://www.mdpi.com/article/10.3390/ijms26031096/s1.

## Abbreviations

CDs	Cytoplasmic droplets
ECM	Extracellular matrix
GO	Gene Ontology
KEGG	Kyoto Encyclopedia of Genes and Genomes
MCODE	Molecular Complex Detection
DIA	Data independent acquisition
ROC	Receiver operating characteristic
ITGAL	Integrin subunit alpha L
ITGB4	Integrin β4
FMNL1	Formin-like 1
INS	Insulin
