# Supplementary figures and images for: Multi-Omics Sequencing Dissects the Atlas of Seminal Plasma Exosomes from Semen Containing Low or High Rates of Sperm with Cytoplasmic Droplets
Source: Int J Mol Sci. 2025 Jan 27;26(3):1096. doi: 10.3390/ijms26031096 (PMC11817786; doi:10.3390/ijms26031096)

SUPPLEMENTARY INFORMATION

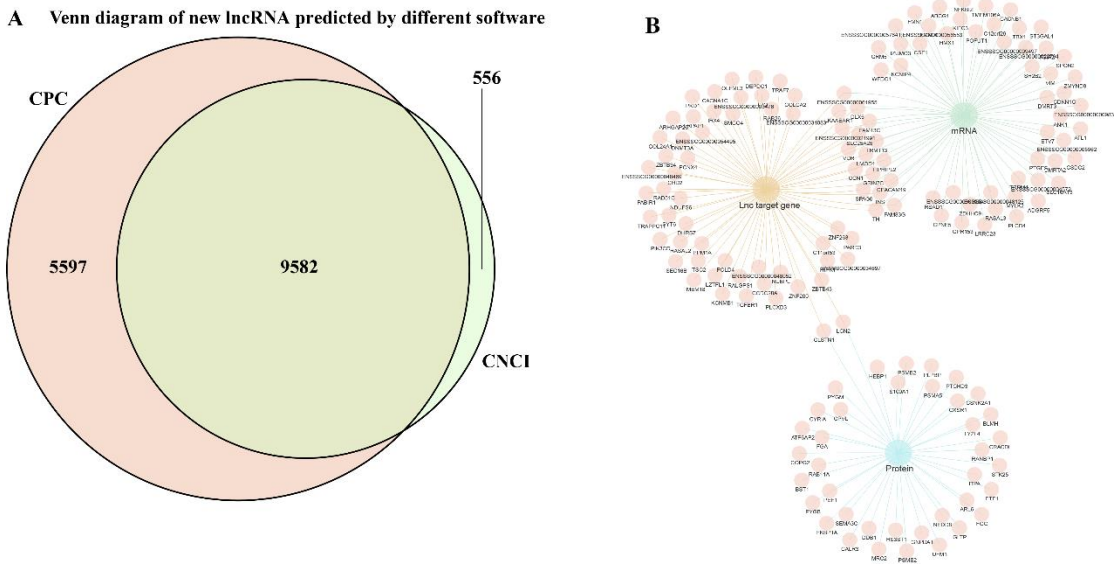

Supplement: Supplementary file 1 [file ijms-26-01096-s001.zip › Supplementary Figures.pdf]
